# Conditionally reprogrammed normal and primary tumor prostate epithelial cells: a novel patient-derived cell model for studies of human prostate cancer

**DOI:** 10.18632/oncotarget.13937

**Published:** 2016-12-21

**Authors:** Olga A. Timofeeva, Nancy Palechor-Ceron, Guanglei Li, Hang Yuan, Ewa Krawczyk, Xiaogang Zhong, Geng Liu, Geeta Upadhyay, Aleksandra Dakic, Songtao Yu, Shuang Fang, Sujata Choudhury, Xueping Zhang, Andrew Ju, Myeong-Seon Lee, Han C. Dan, Youngmi Ji, Yong Hou, Yun-Ling Zheng, Chris Albanese, Johng Rhim, Richard Schlegel, Anatoly Dritschilo, Xuefeng Liu

**Affiliations:** ^1^ Department of Oncology, Lombardi Comprehensive Cancer Center, Georgetown University, Washington, DC, USA; ^2^ Department of Pathology and Laboratory Medicine, Lewis Katz Medical School, Temple University, Philadelphia, PA, USA; ^3^ Department of Pathology and Center for Cell Reprogramming, Georgetown University Medical Center, Washington, DC, USA; ^4^ Beijing Genome Research Institute, Shenzhen, Guangdong, China; ^5^ Department of Biostatistics, Bioinformatics, and Biomathematics, Georgetown University, Washington, DC, USA; ^6^ Department of Radiation Medicine, Georgetown University Medical School, Washington, DC, USA; ^7^ Department of Biomedical Science, Cheongju University, Sandang-Gu, Cheongju Shi Chungbuk, Republic of Korea; ^8^ Department of Pathology, University of Maryland, Baltimore, MD, USA; ^9^ Department of Surgery, Center for Prostate Disease Research, Uniformed Services University of the Health Sciences, Bethesda, MD, USA

**Keywords:** prostate cancer, conditional cell reprogramming, cell lines, patient-derived, cancer cell models

## Abstract

Our previous study demonstrated that conditional reprogramming (CR) allows the establishment of patient-derived normal and tumor epithelial cell cultures from a variety of tissue types including breast, lung, colon and prostate. Using CR, we have established matched normal and tumor cultures, GUMC-29 and GUMC-30 respectively, from a patient's prostatectomy specimen. These CR cells proliferate indefinitely *in vitro* and retain stable karyotypes. Most importantly, only tumor-derived CR cells (GUMC-30) produced tumors in xenografted SCID mice, demonstrating maintenance of the critical tumor phenotype. Characterization of cells with DNA fingerprinting demonstrated identical patterns in normal and tumor CR cells as well as in xenografted tumors. By flow cytometry, both normal and tumor CR cells expressed basal, luminal, and stem cell markers, with the majority of the normal and tumor CR cells expressing prostate basal cell markers, CD44 and Trop2, as well as luminal marker, CD13, suggesting a transit-amplifying phenotype. Consistent with this phenotype, real time RT-PCR analyses demonstrated that CR cells predominantly expressed high levels of basal cell markers (KRT5, KRT14 and p63), and low levels of luminal markers. When the CR tumor cells were injected into SCID mice, the expression of luminal markers (AR, NKX3.1) increased significantly, while basal cell markers dramatically decreased. These data suggest that CR cells maintain high levels of proliferation and low levels of differentiation in the presence of feeder cells and ROCK inhibitor, but undergo differentiation once injected into SCID mice. Genomic analyses, including SNP and INDEL, identified genes mutated in tumor cells, including components of apoptosis, cell attachment, and hypoxia pathways. The use of matched patient-derived cells provides a unique *in vitro* model for studies of early prostate cancer.

## INTRODUCTION

Prostate cancer is the most frequently diagnosed solid malignancy in American men with an estimated 220,800 new cases and 27,540 deaths in 2015 [1]. More than 90% of prostate cancer-related mortality results from widespread metastatic cancer, which initially respond to androgen-deprivation therapy, but subsequently become androgen-independent [2, 3].

In contrast to other cancer types, *in vitro* cultures of human prostatic cells have been limited in availability and scope. Three frequently used spontaneously established cell lines, PC-3, DU145 and LNCaP, all derived from metastases, do not span the range of prostate cancer phenotypes and are not representative of primary adenocarcinomas of the prostate [4]. Patient-derived xenograft (PDX) models are often easier to establish from aggressive, high-grade and metastatic tumors as compared to primary tumors that are slow growing and likely non-metastatic [5–7]. Development of a PDX model can take anywhere from 2 to 12 months with engraftment rates typically from 2% to 50% depending on the tumor type. This limits the ability to use such cancer cell lines and PDXs for predicting responses to drug-, radiation-, or immuno-therapies. Progress in the field has been hindered by the absence of appropriate models of human-derived prostate cancer cells, precluding investigation of transforming alterations and development of treatment approaches. For this reason, primary cultures of malignant prostatic cells and normal, preferably donor-matched, epithelial counterparts grown under identical conditions are needed.

Over the past 20 years, many of the technical hurdles involved in growing primary cultures of human prostatic epithelial cells have been overcome, and a variety of methods have been reported for epithelial cell cultures from radical prostatectomy specimens [4]. However, a lingering question relates to the types of cells grown from prostatectomy specimens and whether they can appropriately represent the epithelial components of normal and tumor prostate tissues. *In vitro*, tumor drug sensitivity screening requires cellular models that are clinically relevant and potentially useful for providing information for the optimization of drug treatment. The normal prostate gland epithelium contains three primary differentiated cell types: luminal, basal and neuroendocrine cells [8]. Luminal cells are columnar epithelial cells that express secretory proteins including prostate-specific antigen (PSA) and markers, such as cytokeratin 8 (KRT8), KRT18, NKX3.1, and high levels of androgen receptor (AR). Basal cells are localized beneath the luminal layer and express markers including KRT5, KRT14 and TP63, but express low levels of AR. A rare third cell type, of neuroendocrine origin, expresses endocrine markers such as synaptophysin and chromogranin A, and does not express AR. There is evidence in support of a basal stem cell population in the prostate [9]. In particular, subpopulations of basal cells, isolated using cell-surface markers, display bi-potentiality and self-renewal in explant culture and tissue grafts [10, 11]. Luminal cells were considered as precursors of human prostate adenocarcinoma based on cancer histological characterization showing expansion of luminal cells and disappearance of basal cells. However, Goldstein et al. showed that histological characterization does not correlate with the cellular origin of the disease and that basal cells can form a prostate tumor in immunodeficient mice with histological and molecular characteristics of prostate cancer [12]. In the mouse model, both luminal and basal cells seem to be the targets for transformation and they induce different phenotypes [13]. In primary human cancer, the luminal phenotype is typical, with glands, AR positivity, and absence of basal cells. However, there is a difference between the outcome phenotype and the actual cell that was transformed [14]. Therefore, basal cells can give rise to tumors of luminal phenotype and basal cells seem to be the efficient target for transformation [11–15].

Recently, exciting technological developments have changed the landscape of possibilities for generating *in vitro* human cancer models. These include 2D conditional reprogramming (CR) cultures [16, 17], as well as 3D organoid cultures [18–25]. Organoid culture models work well for normal prostate cells and advanced prostate cancers [26–28], and the CR technology additionally allows cultures to be established from primary tumors. CR cells cultured from normal epithelium are morphologically undifferentiated and express adult stem cell markers, but can fully differentiate when placed into *in vivo* or *in vitro* conditions that mimic their natural environment [17]. Using CR technology, we were able to identify a patient-specific drug therapy for a rare disease, aggressive recurrent respiratory papillomatosis [29], and others have used the technique for studies of targeted therapy-resistant lung cancer [30], for prostate [31–33] and other types of epithelial cells [34–37].

Previously we generated donor-matched normal/tumor cell lines from a variety of tissue types including breast, lung, colon, and prostate specimens using the CR technology [16, 17]. These included 7 matched normal and tumor prostate CR cell cultures, of which tumor-derived cultures referred to as GUMC-30 in this study (GUMC-29 are matched normal cells) retained tumorigenic potential in SCID mice. These novel cell strains were established from normal and tumor tissues from the same patient without introduction of viral and/or cellular genes. In this study we demonstrate that both, normal and tumor prostate epithelial cells, GUMC-29 and GUMC-30, proliferate indefinitely in CR conditions and predominantly express markers of basal cells in 2D (2-dimensional) culture. However, the tumor cells exhibit an increase in a number of luminal markers when established as xenografts in mice, therefore, further suggesting that the basal-like cell population serves as the origin for prostate tumor, in agreement with previous reports [38, 39]. Exome DNA sequencing of the matched normal and tumor pairs reveals significant differences in several signaling pathways, some of which correspond to those discovered in comprehensive analyses of genetic changes in primary prostate cancer specimens. The ability to rapidly establish cell lines from both normal and cancer prostate biospecimens provides a unique platform for identifying the genetic and molecular events of early prostate cancer and will hopefully enable the development of new approaches for early therapeutic intervention.

## RESULTS AND DISCUSSION

### Growth properties of the matched normal and tumor CR cells (GUMC-29 and GUMC-30 cells)

**Figure 1:**
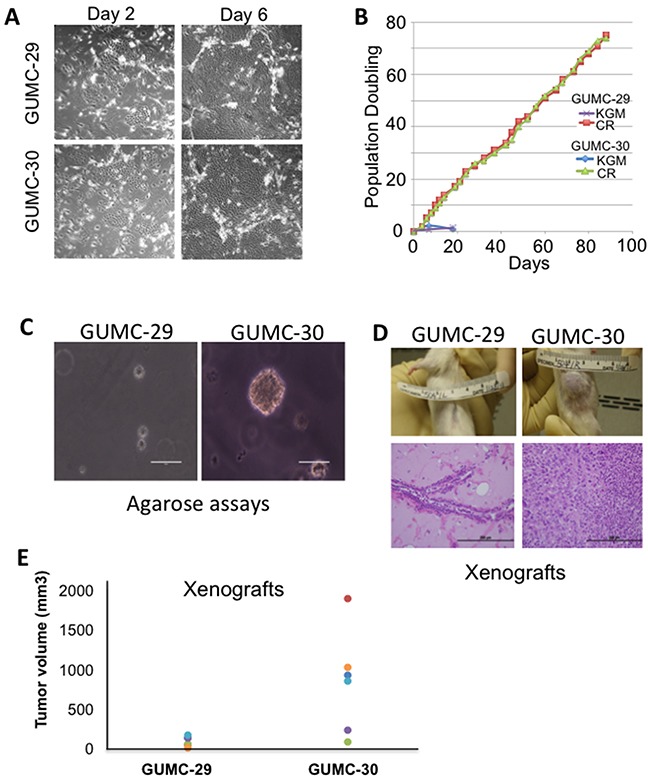
Establishment and biological characterizations of cell cultures from matched normal and tumor prostate specimens **A**. Prostate tissues from a patient with Gleason7 prostate cancer were harvested and digested with trypsin-collagenase, as described previously [16, 17, 61]. The initial cultures were established in keratinocyte growth medium (Invitrogen) and then were plated on a feeder layer of irradiated (3000 rad) Swiss 3T3 cells (J2 subclone) and grown in F medium containing 10 μmol/L ROCK inhibitor (Y-27632). Small colonies were observed after 2 days (left panel). At day 6 (right panel), the typical large islands of epithelial cells were observed that compressed the surrounding feeder cells. **B**. The prostate cells were passaged under CR conditions (F medium containing feeders and Y-27632) or in KGM. Total cell number was recorded at each passage, and the population doubling rate was determined. Only cells grown under CR conditions continued to proliferate past day 20. **C**. Growth in soft agar. GUMC-29 and GUMC-30 CRs were plated in 0.3% agarose in conditioned media and overlaid with 0.6% agar. Only tumor CRs were able to form viable colonies. **D**. GUMC-29 and GUMC-30 were mixed with matrigel and injected subcutaneously into adult male SCID mice. Histopathology of the tumor sample exhibited well differentiated human prostate cancer. **E**. Tumor volumes are shown as measured using verneir calipers. GUMC-29 and -30 cells were grown as spheres for 5-days and then injected on the opposite flanks of nude athymic mice. Paired T-test p-value was 0.018.

Cells of non-malignant and malignant regions of a prostatectomy specimen were grown in keratinocyte serum-free medium (K-SFM) and then transferred to CR conditions to generate immortalized cultures as previously described [8]. The normal CR cells are now designated as GUMC-29 and the tumor CR cells GUMC-30. When grown under CR conditions, both GUMC-29 and GUMC-30 exhibited the typical morphologic characteristics of epithelial cells and were microscopically indistinguishable (Figure 1A). In addition to having the same morphologic appearance, both normal and tumor cells displayed identical growth rates and did not show any evidence of slowed growth or senescence (Figure 1B). However, the GUMC-30 cells formed large colonies in agarose, indicating their anchorage-independent growth properties (Figure 1C). Furthermore, when injected subcutaneously into immunodeficient mice, the GUMC-30 cells formed large tumors within 2 weeks. The tumor size induced by the GUMC-30 cells ranged from 220-2000mm^3^ (Figure 1E) with one animal not forming a tumor. Histopathologic examination revealed poorly differentiated adenocarcinoma (Figure 1D). The GUMC-29 cells did not grow is soft agar and did not form xenograft tumors (Figures 1C-E).

To confirm the human origin of the xenograft and its relationship to the injected GUMC-30 cells, we performed STR analysis (Table 1). Both the GUMC-29 and GUMC-30 cultures showed identical STR patterns as did the GUMC-30-induced xenograft tumors. There are no cell lines in the ATCC database that match the STR results shown.

**Table 1:** STR analysis of prostate GUMC-29 and GUMC-30 cells

Cell ID	GUMC-29		GUMC-30		GUMC-30 xenograft		
MARKER	Allele1	Allele2	Allele1	Allele2	Allele1	Allele2	ATCC MATCH
AMEL	X	Y	X	Y	X	Y	**no match**
CSF1PO	10	12	10	12	10	12	
D13S317	12	14	12	14	12	14	
D16S539	11	12	11	12	11	12	
D18S51	12	15	12	15	12	15	
D19S433	14	17.2	14	17.2	14	17.2	
D21S11	29	30	29	30	29	30	
D2S1338	18	25	18	25	18	25	
D3S1358	17	18	17	18	17	18	
D5S818	9	12	9	12	9	12	
D7S820	9	11	9	11	9	11	
D8S1179	12	13	12	13	12	13	
FGA	18	26	18	26	18	26	
TH01	6	9.3	6	9.3	6	9.3	
TPOX	9	11	9	11	9	11	
vWA	14	17	14	17	14	17	

We interpret these results as that CR conditions allow immortalization of both normal and tumor primary prostate cells while preserving their tumorigenic potential.

### Different properties of the matched normal and tumor CR cells compared to cancer cell lines

**Figure 2:**
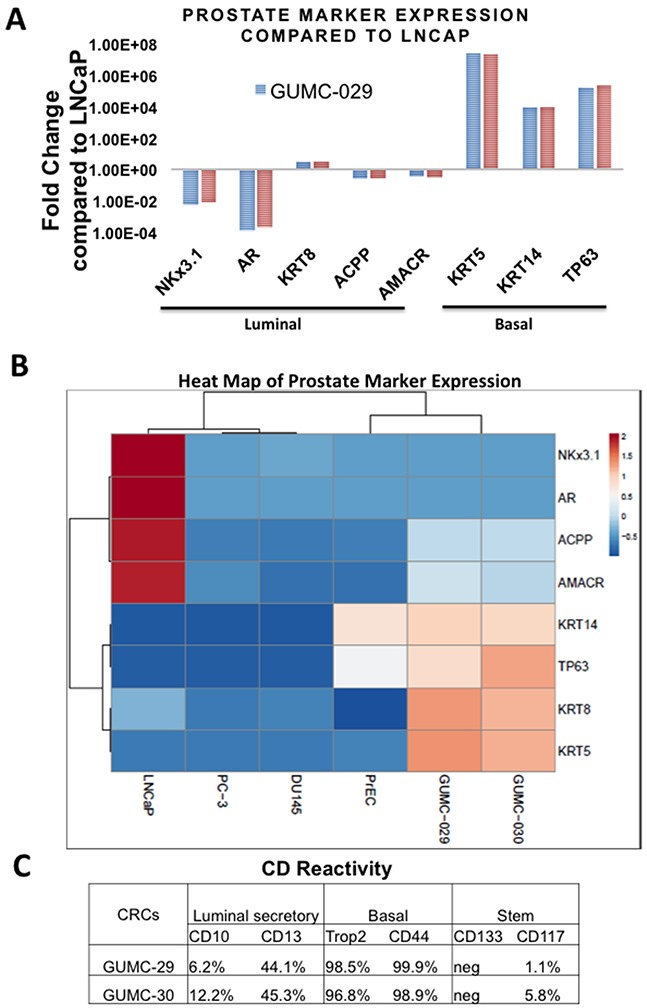
Characterization of prostate epithelial cells markers using qRT-PCR and Flow Cytometry **A**. Results of quantitative Reverse Transcription PCR (qRT-PCR) performed on total RNA extracted from 3 biological replicas of CR prostate cultures compared to LNCaP for basal cell markers: KRT5, KRT14, TP63 and luminal cell markers: AR, AMACR, ACPP, NKX3.1, and KLK3. **B**. Heatmap of prostate marker expression determined by qRT-PCR in GUMC-29/30, LNCaP, PC3, DU145, and PrEC. **C**. The number of cells expressing markers of luminal secretory, basal, and stem cells was measured using flow cytometry.

To evaluate prostate marker expression in GUMC-29 and GUMC-30, we utilized qRT-PCR and flow cytometry techniques. Compared to the established cancer cell line LNCaP, both CR lines expressed high levels of basal cell markers KRT14, KRT5 and TP63, low to negligible levels of luminal cell markers such as AR and NKX3.1, and similar levels of other luminal markers such as AMACR, ACPP and KRT8 (Figure 2A). Importantly, other spontaneously immortalized prostate lines, DU145 and PC3, do not express basal cell markers TP63, KRT5, and KRT14 and are similar to LNCaP cells in this respect (Figure 2B). However, these cells express very low levels of luminal cell markers AR and NKX3.1 comparable to GUMC-29/30. Interestingly, AMACR, ACPP, and KRT8 levels are lower in PC3 and DU145 compared to GUMC-29/30. Analysis of prostate marker expression in the primary prostate cells PrEC showed that these cells express high levels of basal markers TP63 and KRT14, but very low levels of all luminal markers. These data suggest that both phenotypes, basal and luminal are present in CR cultures. Notably, there are no significant differences in the levels of expression of any prostate differentiation markers between GUMC-29 and GUMC-30, suggesting that the normal and tumor cells were inherently similar or that the CR conditions converted them to a similar and less-differentiated state.

### CR cells are “transit-amplifying” cells

Flow cytometry analysis of surface CD markers in GUMC-29/30 demonstrated that nearly all normal and tumor cells express basal cell markers, CD44 and Trop2 (CD49f) (Figure 2C and Supplementary Figure 1). Interestingly, about 45% of the normal and tumor cells also expressed the luminal CD13 marker, while only a small portion (6-12%) expressed the CD10 luminal marker. We found a population of CD117-expressing cells in the prostate cultures, considered to be prostate stem cells [10].

Approximately 5% cells of the GUMC-30 cancer cell population were CD117-positive, while only 1% of the GUMC-29 normal cells were CD117-positive. No CD133-positive cells were detected in CR cultures.

Taken together, the results of Flow Cytometry and RT-PCR analyses suggest that cells growing under CR conditions resemble “transit-amplifying” cells, which express markers of both basal and luminal cells and can differentiate into either phenotype upon receiving an appropriate stimulus [40–42]. Using skin and tracheal cells, we have previously shown that transit-amplifying-like cells from CR cultures can ultimately differentiate into mature epithelium containing both basal and luminal cells [43].

### Differences in gene expression between normal and tumor CR cells

**Figure 3:**
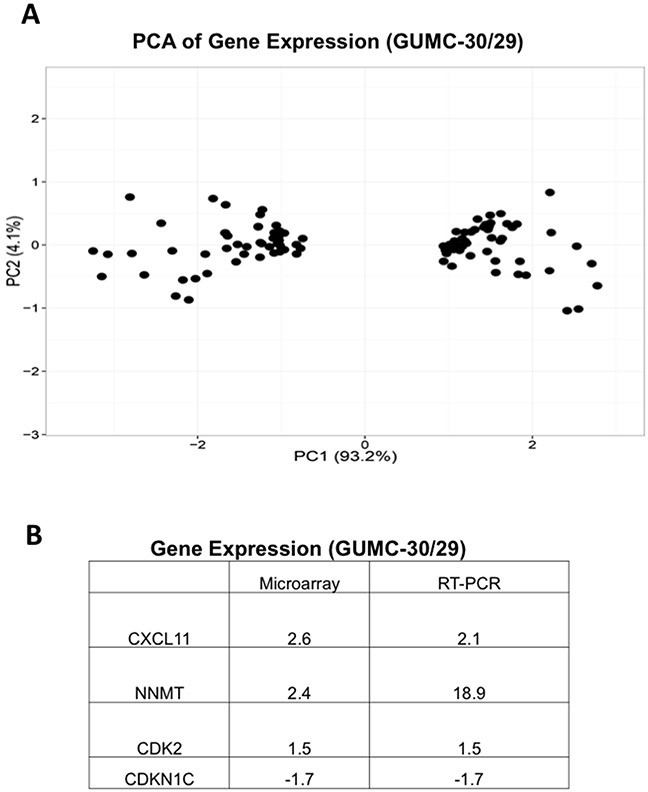
Differential gene expression in GUMC-29 and GUMC-30 **A**. PCA analysis of differential gene expression in normal and tumor CR cells detected by microarray. **B**. Differential expression of selected genes was confirmed using qRT-PCR analyses.

To study differences in gene expression, we performed Affymetrix GeneChip microarray. We identified 87 genes differentially regulated in GUMC-30 compared to GUMC-29 (Figure 3A and Supplementary Data Set 1). Genes involved in cellular development, growth and proliferation, and metabolism were identified. RT-PCR confirmed differential expression of selected genes (Figure 3B). Some of the genes may play a role in prostate tumorigenesis, for example CXCL11 (Chemokine (C-X-C Motif) Ligand 11), NNMT (Nicotinamide N-methyltransferase) and CDK2 have been reported to be over-expressed in prostate cancer and CDKN1C is repressed in breast and prostate cancer [44–47]. Further studies will investigate the role of these proteins in prostate tumorigenesis.

### Differential sensitivity of matched CR cells to docetaxel

To test the patient-derived CR cells for potential use for drug screening, we exposed matched CR cells to a commonly used chemotherapeutic agent, docetaxel. As shown in Supplementary Figure 2, the IC50 values of docetaxel to GUMC-29 and GUMC-30 were 1.79 μM and 0.29 μM, respectively. These data suggested that the matched normal and tumor CR cells can be used to aid drug selection for the individual patients or as a system that enables high throughput drug discovery. Indeed, we were able to individualize drug therapy for a patient with an aggressive lung tumor using CR technology [29]. Others have used our technique for studying and developing therapies for targeted therapy-resistant lung cancer [30]. Most recently, our technology has been used to screen for new drugs or to discover new targets [33, 48–50].

### Re-expression of androgen receptor in normal and tumor CR cells under differentiation conditions

**Figure 4:**
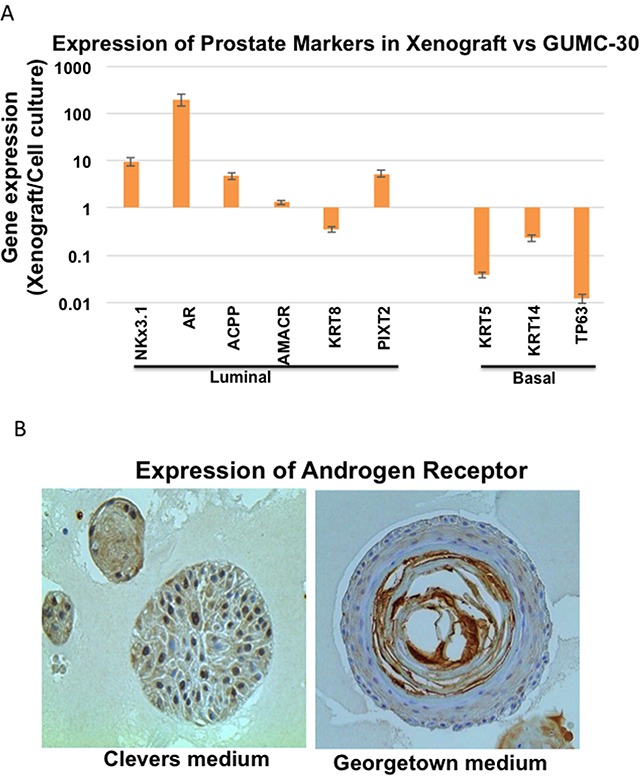
Differential gene expression in tumor CRs and xenografted tumor **A**. Expression of luminal and basal epithelial markers were measured using qRT-PCR in GUMC-30 cultured under CR conditions and GUMC-30 xenograft tumors. Expression of most luminal markers increased in xenograft tumor compared to CRs, while basal markers significantly decreased in the tumor xenograft compared to CR. **B**. Organoid cultures were established as described in Materials and Methods. Organoids were fixed overnight with 4% paraformaldehyde at room temperature and the following day with 70% ethanol. Fixed organoids were embedded in paraffin for sectioning at 5 μm before probed with anti-AR (Santa Cruz, sc-816) for immunohistochemistry. Images of growing organoids were acquired using EVOS FL Cell Imaging System (Invitrogen) for microscopic imaging.

Since GUMC-30 cells formed adenocarcinoma when injected into immunodeficient mice (Figure 1D, E), we hypothesized that this process may be accompanied by changes in prostate marker expression. qRT-PCR assays demonstrated that luminal cell markers, including AR and NKX3.1, were up-regulated 10- to 100-fold in the xenograft-derived tissues when compared to the GUMC-30 adherent cell cultures (Figure 4). In contrast, basal cell markers, including TP63, KRT14, and KRT5, dramatically decreased in the xenografts (Figure 4A). These data indicate that the tumorigenic GUMC-30 cells are somewhat plastic in their expression profiles and that the *in vivo* xenograft conditions favor a more differentiated, luminal-like cell phenotype. These data further support previous observations that basal/trans-amplifying cells may form prostate adenocarcinomas in immunodeficient mice [51–55], and, therefore, represent important target cells in prostate cancer.

As shown in Figure 4B, normal CR cells from PrEC (Lonza) also re-expressed androgen receptor when they were cultured in organoid conditions with both Clevers’ medium and Georgetown medium (CR medium or F medium with Y-27632).

These data suggested that both, normal and tumor CR cells, maintain some differential potential and their original benign and malignant phenotypes in *in vitro* or *in vivo* differentiation environments.

### Karyotype and exome sequence analysis of the matched normal and tumor CR cells

**Figure 5:**
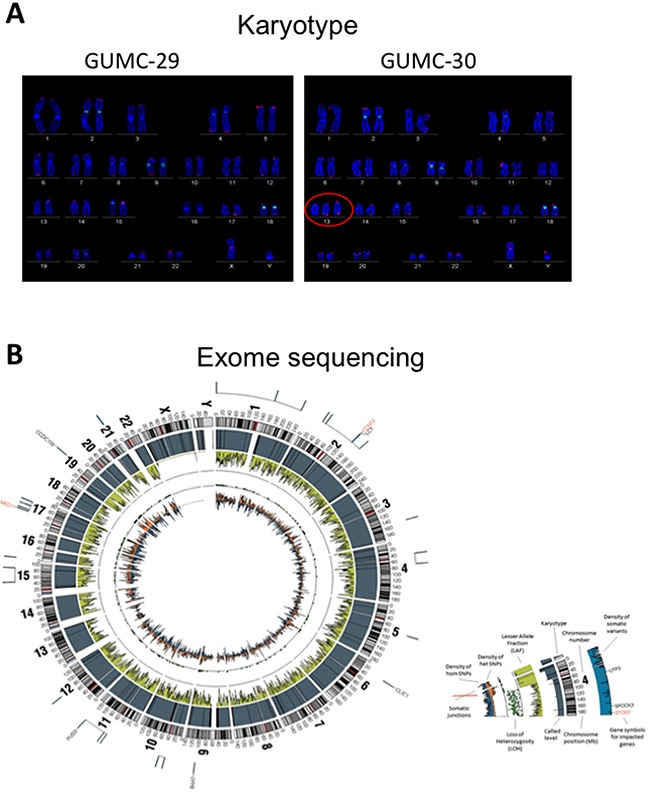
Karyotype and exome sequence analysis of the prostate CR cells Both GUMC-29 and GUMC-30 CRs were subjected to karyotyping and exome-sequencing analyses. **A**. Roughly 10% of population of GUMC-30 cells exhibited trisomy at chromosome 13, while the remainder of GUMC-30 cells and 100% GUMC-29 harbor normal karyotypes. **B**. Exome-sequencing was performed on both GUMC-29 and GUMC-30 using the CG platform as showing a Circos visualization of variations detected in the Genome, along with other associated data. Density of hom SNPs represents the density of high confidence homozygous SNPs in 1Mb windows, arbitrarily scaled in a histogram with the Y-axis pointing inward; Density of het SNPs represents the density of heterozygous SNPs in 1Mb windows, arbitrarily scaled in a histogram with Y-axis pointing outward; Lesser Allele Fraction (LAF) represents the single-sample LAF estimate for 100 kb windows, with Y-axis scale of 0 to 0.5, pointing inward. Estimates are based on read counts at called heterozygous loci; Called Ploidy represents the called ploidy from a segmentation file, which partition the reference genome into regions of distinct ploidy levels, giving the estimated ploidy, the average and relative adjusted coverage for each segment, and measures of confidence in the called segments. They are arbitrarily scaled with the Y-axis pointing inward; Karyotype represents the standard Circos ideogram depicting chromosome position and chromosome number.

**Figure 6:**
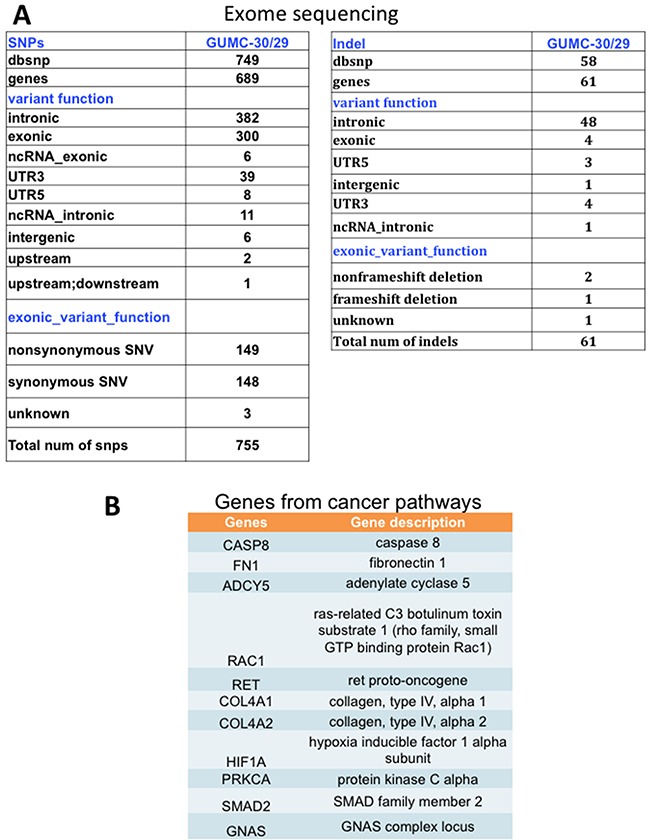
SNPs and INDELs and pathway analysis in tumor CR cells **A**. Numbers of SNP and INDELs, plus pathways, that differ in GUMC-30 versus GUMC-29. **B**. KEGG pathway analysis of cancer pathways genes.

**Figure 7:**
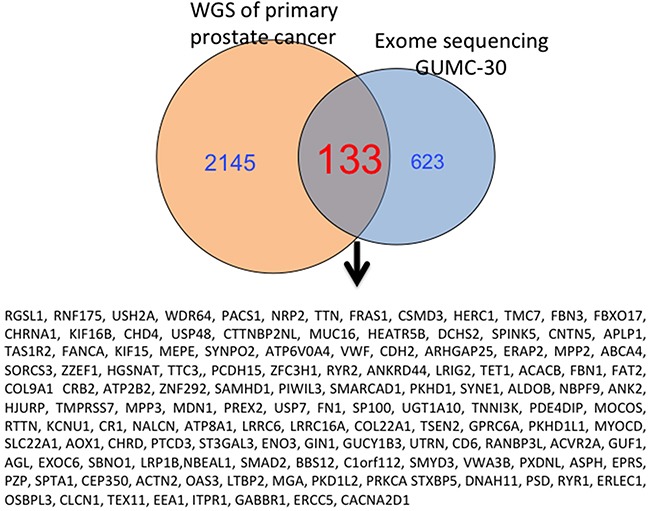
Overlapping genes from GUMC-30 and Garraway's study with NGS are shown 133 Mutated genes in tumor CR cells were presented in result from a previous study with NGS from the patients with primary prostate cancer.

To further characterize these matched prostate CR cells at the cytogenetic and molecular levels, we performed karyotype analyses followed by exome sequencing, including SNPs and INDELs. The karyotype analyses revealed that approximately 10% of the GUMC-30 cells carry an additional copy of chromosome 13 (Figure 5A). We generated a Circos plot of the variants detected by sequencing analysis (Figure 5B). Our exome sequencing analysis has identified a total of 815 variants present only in the GUMC-30 sample. These variants were found to associate with 756 genes with unique HGNC gene symbols. Consensus genotype calls were generated using the UnifiedGenotyper tool from GATK (v3.5) and annotated using the Annovar package. Different basic types of annotations were separated into several tables based on the effects of a variant on gene, transcript, and protein sequence, such as an amino acid change, frameshift, or promoter region alteration, etc. (Figure 6A) based on GRCh37 (Supplementary Data set 2). We compared our variant gene list with the publicly available 2755 genes prostate cancer list [56]. Using Venn diagram analysis we identified a set of 133 genes common to the both lists (Figure 7) among which 11 genes belong to the KEGG cancer pathway gene set (Figure 6B, Supplementary Data Set 3), including FN1, CASP8, HIF1a, SMAD2, RAC1, and RET [51–55].

Genomics is a useful tool for cancer research that has largely focused on basic mutational catalogs in primary tumors. Given the complexity of primary prostate cancer, it is necessary to deepen the structural characterization of cancer genomics and comprehensive functional characterization of cancer cells. This will help further understanding of basic and translational biology of human prostate cancer. The matched normal and tumor CR cells from the same patients provide a novel functional platform for these studies.

### Selection of tumor CR cells from mixed normal and tumor population

Considering that CR technique supports growth of both normal and tumor cells and that all clinical tissue specimens are mixtures of normal, tumor and stromal cells, we wanted to select out pure tumor cells from mixed cultures.

**Figure 8:**
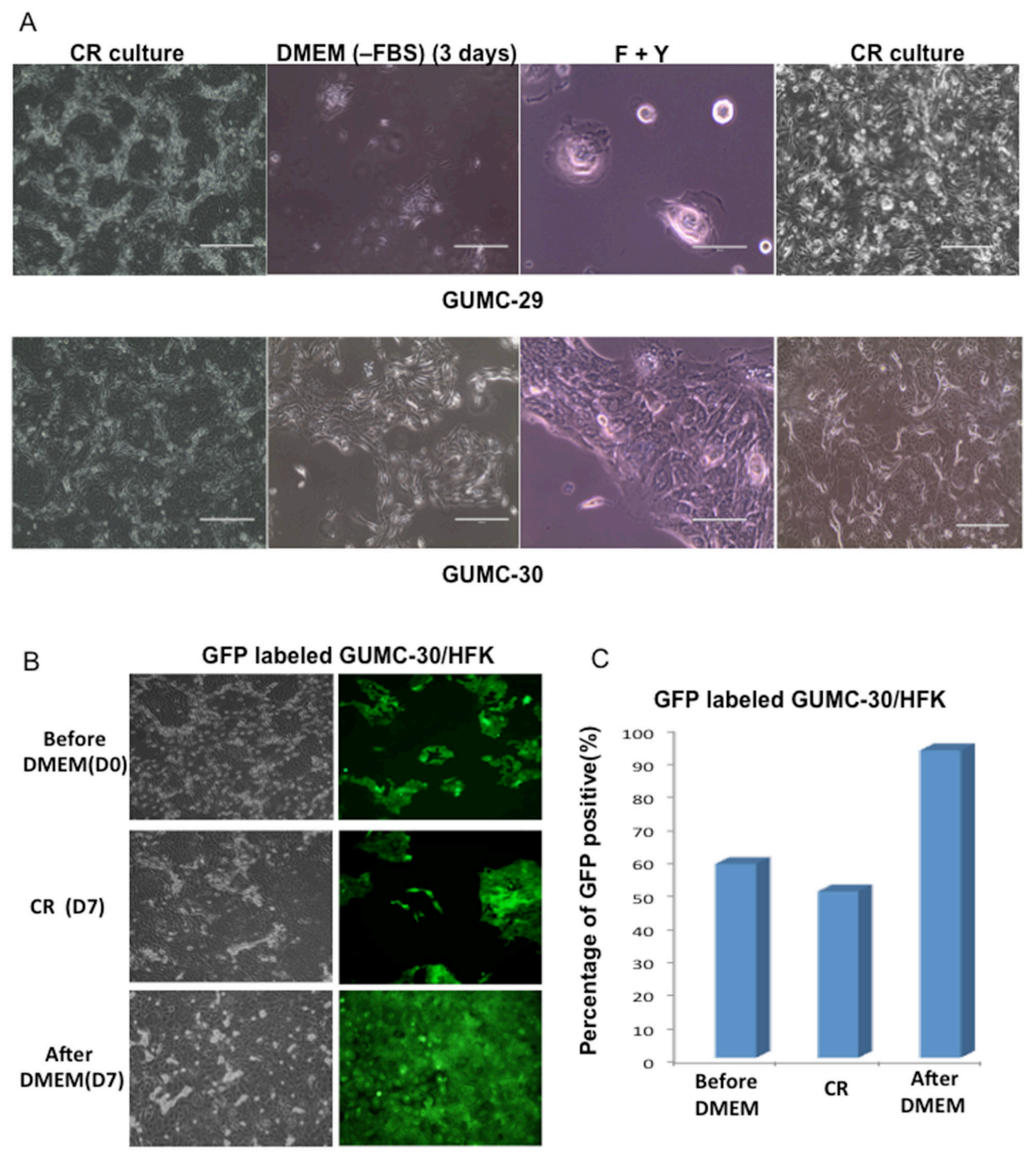
Selection of Tumor CR cells GUMC-29 and -30 under CRC condition were changed to DMEM without FBS for 3 days and media were replaced with F medium plus Y-27632 for 5 days, lastly leftover were re-plated in CRC condition **A**. normal CRCs (GUMC-29) entered complete senescence stage without any recovery once the cells were transferred back to CR condition while tumor CRCs (GUMC-30) were able to recover from selection process and formed typical epithelial colonies as usual The mixed culture with GFP-labeled GUMC-30 and non-labeled normal foreskin keratinocytes (HFK) (50:50) underwent the same selection protocol, only GFP positive cells presented in culture after selection **B** and **C**.

Various culture conditions were tested to identify factors that allowed proliferation of tumor cells, but did not support expansion of normal cells. We found that culturing CRs in DMEM without serum for 3 days led to differentiation of normal GUMC-29 cells, while supporting a “fibroblast”-like or mesenchymal morphologic phenotype of GUMC-30 (Figure 8A). Interestingly, CR conditions reversed “fibroblast”- or mesenchymal-like cells back to epithelial morphology. To further validate this approach, GFP-labeled GUMC-30 cells were mixed with normal HFKs in equal amounts and were first grown in DMEM without serum for 7 days. Only “green” tumor CR cells grew after 7 days of selection (Figure 8B, 8C).

This selection procedure may be particularly important for primary cancer studies. Currently, CR technology is the only method by which expansion of primary prostate cancer cells is obtained. The organoid protocol can be used for establishing cultures from normal mouse and human prostate tissue with efficiency > 95%. However, advanced prostate cancers yield only about 15 – 20% efficiency [26–28]. Growing organoid cultures derived from primary prostate cancers has not been successful, possibly due to overgrowth by normal prostate epithelium present within each sample [26–28].

If this is truly the case, pre-selection procedures will be important. We are currently working to develop selection methods that allow “purification” of tumor for selective expansion as CR cells with different approaches summarized in a recently accepted paper by Nature Protocols [17].

### Micro-heterogeneity

Finally, given that primary prostate cancer is highly heterogeneous disease, it is important to establish a functional method to study tumor heterogeneity. We used a tissue sample collected from a patient with primary prostate cancer at Georgetown Lombardi Cancer Center for preparing a single cell suspension that was diluted to 1 cell/200 μl FY medium, and plated 100 μl volumes per well of ten 96-well plates. We established 9 clones from single cell cultures using CR technology. Our data suggests that clones have different invasive abilities (Supplementary Figure 4). Comprehensive genomics and functional studies (especially response to drugs) on these clones will help further understanding heterogeneity of primary prostate cancer.

### Summary

We have generated matched normal and tumor cell cultures from a patient with primary prostate adenocarcinoma. These cultures, while exhibiting similar morphologies and division rates in CR conditions, were different in their ability for anchorage-independent growth and capacity to induce tumor formation in immunodeficient mice. The normal and tumor cells also displayed differences in karyotype, gene expression, and exome sequences. Some of the genetic mutations have been noted in previous comprehensive analyses, including genes involved in pathways that regulate cell attachment, cell apoptosis, and HIF signaling. We provide a novel functional platform for cancer biology, discovery of biomarkers and anti-cancer drugs, and cancer precision medicine.

## MATERIALS AND METHODS

### Generation of primary cell cultures

The non-malignant tissue (RC-123N) and malignant tissue (RC-123T) used for generating the primary cells were obtained from a radical prostectomy of a 57-year old European American patient. Samples were obtained according to an approved Internal Review Board (IRB) protocol from the Walter Reed Army Medical Center and the Uniformed Services University of the Health. This patient was clinically staged with T3b adenocarcinoma with Gleason grade 3+4=7. Non-malignant cells were derived from histopathologically confirmed non-cancerous regions of the prostate. The method for generating primary prostate cell cultures has been previously described [57]. Briefly, the tumor and non-malignant tissues, obtained by an experienced pathologist, were minced into 1-2 mm small fragments with a sterile scalpel. The tissue was then placed into type 1 collagen-treated dishes (Becton-Dickinson, Boston, MA) containing growth medium and were allowed to attach to the bottom surface of the culture dishes. The cells were incubated for approximately one week at 37°C in 5% CO_2_ until reaching semi-confluency. Aliquots of the primary cultures were then frozen and stored in liquid nitrogen. For serial passages, routine trypsinization was performed weekly in collagen-treated culture dishes, with the split ratio of cells being 1:2. The cells were grown in Keratinocyte serum-free medium (K-SFM) supplemented with bovine pituitary extract and recombinant epidermal growth factor (Life Technologies, Inc., Gaithersburg, MD).

### Generation of conditionally reprogrammed cells, GUMC-29 and GUMC-30

Cells were seeded on a feeder layer of lethally irradiated (30 Gy) J2 fibroblasts in F medium. The F medium consisted of 25% Ham's F-12 nutrient mix (Life Technologies) and 75% complete DMEM, supplemented with 25 ng/mL hydrocortisone, 5 mg/mL insulin, 0.1 nmol/L cholera toxin (Sigma-Aldrich, St. Louis, MO), 250 ng/mL Fungizone (Thermo Fisher Scientific, Waltham, MA), 0.125 ng/mL epidermal growth factor, and 10 mg/mL gentamicin (Life Technologies). In most experiments, cells were cultured in the presence of the ROCK inhibitor Y-27632 at a final concentration of 5 μmol/L (Enzo Life Sciences, Farmingdale, NY). In the absence of feeder cells, cells were grown either in F medium containing Y-27632 or in keratinocyte growth medium (KGM) (Life Technologies).

### Passaging CR cells

To remove J2 feeder cells, prostate co-cultures were rinsed with Dulbecco's PBS and treated with 0.05% trypsin-EDTA (Life Technologies) for 30 seconds at room temperature. The culture vessel was then gently rocked until the feeder cells detached, and the feeder cells were removed by aspiration. The prostate epithelial cells were washed with Dulbecco's PBS and treated with trypsin-EDTA for 3 to 5 minutes at 37 C. The prostate epithelial cells were detached by gentle tapping, and trypsin was neutralized by adding Dulbecco's PBS containing 10% fetal bovine serum. After centrifugation, the prostate epithelial cells were suspended in F medium and plated on freshly irradiated feeder cells.

### Selection of tumor CR cells

GuMC-29, GUMC-30 or mixed CR cells with GFP-labeled GUMC-30 and non-labeled normal foreskin keratinocytes (HFK) from above CR condition were initially selected in DMEM without FBS for 3 days, then in F medium with Y-27632 for 5 days. All the leftover were placed back in CR condition.

### Affymetrix microarray analysis

Total RNA was extracted using an RNeasy Kit (Qiagen, Valencia, CA, USA). RNA labeling and hybridization were performed according to Affymetrix standard protocol for one-cycle target labeling. Fragmented cRNA was hybridized in triplicate to Affymetrix GeneChip HG-U133A 2.0 arrays (Affymetrix, Santa Clara, CA). Affymetrix data analysis included pre-processing of the probe-level Affymetrix data (CEL files). We applied RMA for background adjustment, quantile method for normalization, and the ‘median polish’ for summarization. The triplicate arrays representing the same subject were averaged. The random variance model implemented in BRB-ArrayTools (NCI, Bethesda, MD) was used for this analysis [48] Pathway analysis was performed with Database for Annotation, Visualization and Integrated Discovery (DAVID) [58].

### Quantitative RT-PCR

Total RNA was extracted from 3 biological replicates of CR prostate cultures, including GUMC-29, GUMC-30, and established cell lines LNCaP, DU145, PC3 as well as primary PrEC cells using RNeasy kit (Qiagene) as previously described [59]. One microgram of total RNA was reverse-transcribed using the High Capacity cDNA Reverse Transcription Kit (Life Technologies) in 20 μl of reaction mixture. Two microliters of reverse transcription reaction corresponding to 100 ng of total RNA were taken into quantitative real-time PCR (qRT-PCR) reaction that was performed in triplicate using TaqMan Gene Expression Assays (Applied Biosystems) on the Applied Biosystems 7900HT Fast Real-time PCR System using fast mode. Amplification of human β-actin mRNA was used as an endogenous control to standardize the amount of sample added to the reaction. The comparative cycle threshold (CT) method was used to analyze the data by generating relative values of the amount of target cDNA (Applied Biosystems). CT represents the number of cycles for the amplification of target to reach a fixed threshold and correlates with the amount of starting material present. To obtain relative values, the following arithmetic formula was used: 2-ΔCT, where ΔCT = difference between the threshold cycles of the target and an endogenous reference.

### Flow cytometry analysis

CD reactivity of luminal (CD10 and CD13), basal (CD44 and CD49f (Trop2)), and stem cells (CD117 and CD133) was detected using fluorophore-conjugated CD antibodies (Biolegend). Briefly, trypsinized cells, prepared as a single cell suspension, were resuspended in 50 μl aliquots of 0.1% bovine serum albumin-Hanks’ balanced salt solution (BSA-HBSS). Fluorophore-conjugated CD antibodies were added to the cell suspensions (0.1 μg of anti-CD10, CD13, CD117 and CD133 and 0.05 μg of anti-CD44 and CD49f antibody were added) and incubated for 1 hr at room temperature in the dark. Isotype-specific fluorochromated antibodies were used as negative controls to delineate the negative cell population. The reaction was stopped by the addition of 1 ml of 0.1% BSA-HBSS. The cells were centrifuged and fixed in 0.35 ml of 2% paraformaldehyde. Flow analysis was done by a FACScan (Becton Dickinson, Mountain View, CA) machine fitted with a 488 nm laser. Events that registered outside this trace were scored as positive, and 10,000 events were collected for each sample. The percentage of positive events was determined.

### Oganoid cultures

Organoid cultures were set up according to previous protocols [26–28]. Briefly, 120 μl of BD Matrigel® (BD Biosciences, San Jose, CA, Cat# 356230) were added to the bottom of a well 6 well plate (Falcon, Durham, NC, Cat # 353046) and incubate gel at 37°C for approximately 10 min. Next, a second layer of 1.2 ml matrigel containing 50,000 cell suspension was added on top and allow gel at 37°C for additional 15-20 min. Then, 2ml F medium with 10 μM Y-27632 (Georgetown Medium) or Clevers’ medium [26–28] will add on the surface. The medium was changed every two days. Organoids were fixed overnight with 4% paraformaldehyde at room temperature and the following day with 70% ethanol. Fixed organoids were embedded in paraffin for sectioning at 5um before probed with antibodies for immunohistochemistry or immunofluorescence. All samples were stained for H&E and the following antibodies were used for immunostaining: 1:125 dilution of rabbit AR (N20) (Santa Cruz; sc-816) and mouse p63 (Santa Cruz; sc25268) and 1:800 dilution of mouse CK 18 (Cell Signaling, 4548P). Images of growing organoids were acquired using EVOS FL Cell Imaging System (Invitrogen) for microscopic imaging.

### Agarose assay

To assay anchorage-independent growth, 1 ml of 0.3% agarose containing 1 × 10^3^ cells was layered over 1 ml of 0.6% agarose in each well of 6-well plate. A sterile 3% agarose stock solution was prepared in D-PBS and diluted to the required concentrations by mixing with conditioned medium as previously described. Cultures were overlaid with 0.5 ml of this medium and further additions were made, as necessary, to prevent desiccation for a period of 3–4 weeks.

### Tumorigenicity in SCID mice

To determine tumorigenicity, 1×10^7^ cells in 0.2 ml of matrigel were injected subcutaneously into the mid-dorsal intracapular regions of adult male SCID mice. The mice were observed for up to 6 months for tumor development, as previously described [16]. To confirm the tumorigenic capacity of the lines, the following protocol was also used. Normal and tumor cell CRs, grown in the presence of feeder cells and Y-27632 (5 μM) cells, were subsequently plated in conditioned medium and seeded in low attachment plates. Cells were allowed to grow as spheres for 5-days. The media was changed on day 3. Cells were harvested and a single cell suspension was prepared by trysinization of the spheres. 100,000 cells were injected in nude athymic mice. Tumor mass was measured using verneir calipers. Mice were observed for up to one month for tumor development.

### Cytogenetic analysis

Chromosome counts, ploidy distribution, and Giemsa (G)-banded karyotypes were prepared by standard protocol as described previously [60].

### Exome sequencing data analysis

DNAs from GUMC-29 and -30 were extracted and exome-sequencing was performed with Complete Genomics platform. Sequence reads were processed with a pipeline consisting of the following elements: (1) base calls generated in real-time on the Agilent SureSelect system; (2) Perl scripts developed in-house to produce demultiplexed fastq files by lane and index sequence; (3) demultiplexed BAM files aligned to a human reference (hg19) using BWA (Burrows-Wheeler Aligner). Read-pairs not mapping within +2 standard deviations of the average library size (*125+15 bp for exomes) are removed. All aligned read data were subjected to the following steps: (1) duplicate removal” was performed, (i.e., the removal of reads with duplicate start positions; Picard MarkDuplicates); (2) indel realignment was performed (GATK IndelRealigner) resulting in improved base placement and lower false variant calls; (3) base qualities were recalibrated (GATK Table Recalibration).

Variant detection and genotyping were performed using the UnifiedGenotyper tool from GATK (v3.5). Variant data for each sample were formatted (variant call format) as “raw” calls that contain individual genotype data for one or multiple samples, and flagged using the filtration walker (GATK) to mark sites that are of lower quality/false positives, e.g., low quality scores (<= 50), allelic imbalance (>= 0.75), long homopolymer runs (> 3) and/or low quality by depth (QD< 20).

## SUPPLEMENTARY FIGURES








