# Supplementary material for: Conditionally reprogrammed normal and primary tumor prostate epithelial cells: a novel patient-derived cell model for studies of human prostate cancer
Source: Oncotarget. 2016 Dec 21;8(14):22741–58. doi: 10.18632/oncotarget.13937 (PMC5410259; doi:10.18632/oncotarget.13937)
Supplement: Supplementary file 1 [file oncotarget-08-22741-s001.pdf]

Conditionally reprogrammed normal and primary tumor prostate epithelial cells: a novel patient-derived cell model for studies of human prostate cancer

SUPPLEMENTARY FIGURES AND TABLES

A

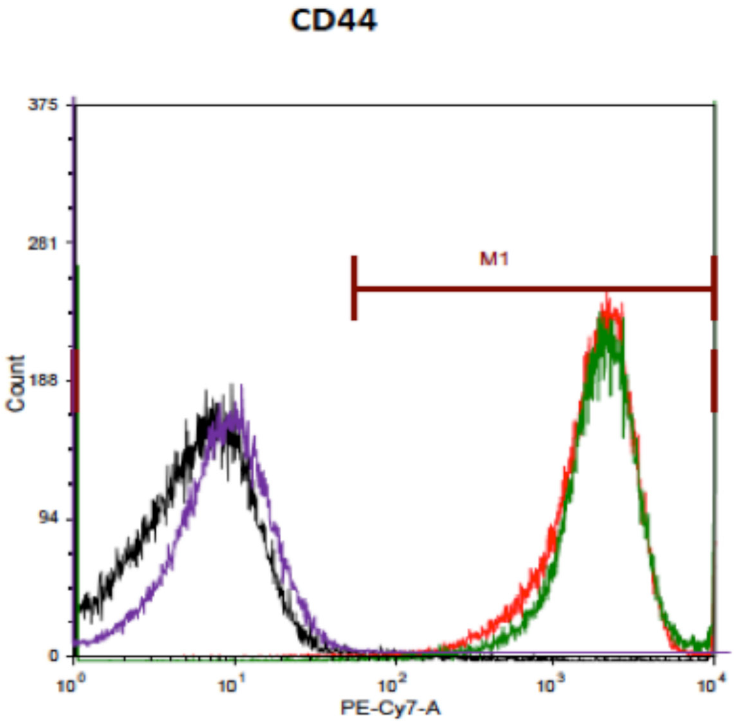

Filename	Low bound	High bound	# of Events	% of gated	Geometric Mean
GUMC-29, Isotype control	55.96	10000.00	1018	1.69	364.09
GUMC-30, Isotype control	55.96	10000.00	1668	3.52	693.09
GUMC-29, CD44	55.96	10000.00	60145	99.85	1695.20
GUMC-30, CD44	55.96	10000.00	46155	98.86	2180.61

Supplementary Figure 1: Expression of cell surface markers measured by Flow Cytometry. A. CD44. (*Continued*)

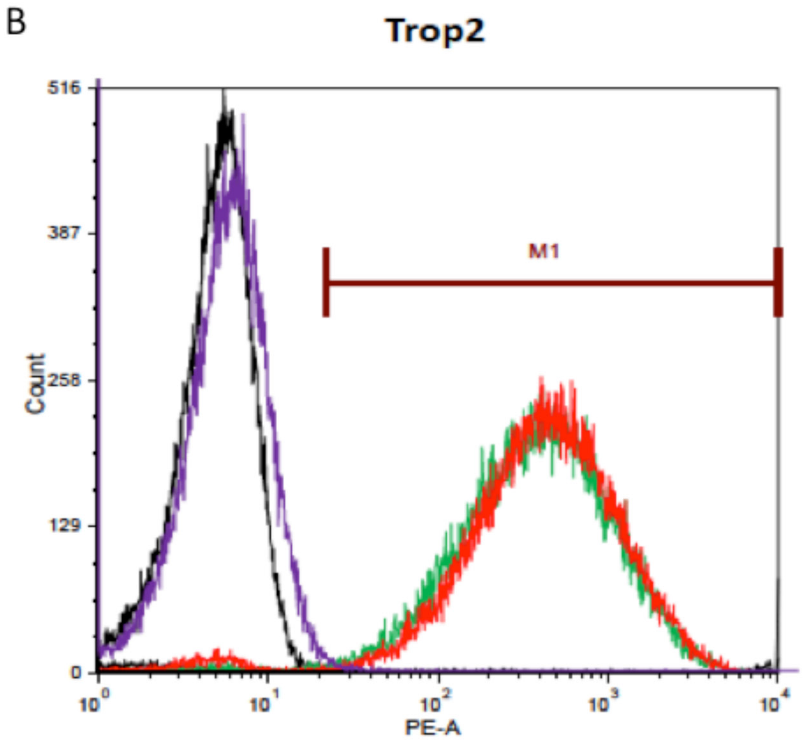

	Filename	Low bound	High bound	# of Events	% of gated	Geometric Mean
(black line)	GUMC-29, Isotype control	21.90	10000.00	1037	1.71	812.16
(purple line)	GUMC-30, Isotype control	21.90	10000.00	210	0.40	509.96
(red line)	GUMC-29, Trop2	21.90	10000.00	45054	98.40	381.06
(green line)	GUMC-30, Trop2	21.90	10000.00	47163	96.72	433.70

Supplementary Figure 1: (*Continued*) Expression of cell surface markers measured by Flow Cytometry. B. Trop2 (*Continued*)

C

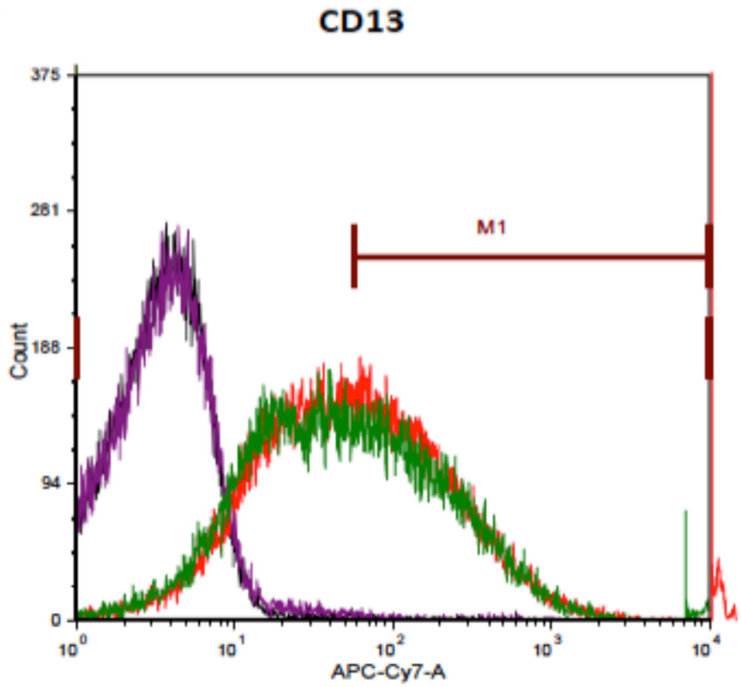

Filename	Low bound	High bound	# of Events	% of gated	Geometric Mean
GUMC-29, Isotype control	55.96	10000.00	361	0.59	2.55
GUMC-30, Isotype control	55.96	10000.00	394	0.31	48.30
GUMC-29, CD13	55.96	10000.00	26414	44.12	2.86
GUMC-30, CD13	55.96	10000.00	21525	45.28	47.71

Supplementary Figure 1: (*Continued*) Expression of cell surface markers measured by Flow Cytometry. C. CD13 (*Continued*)

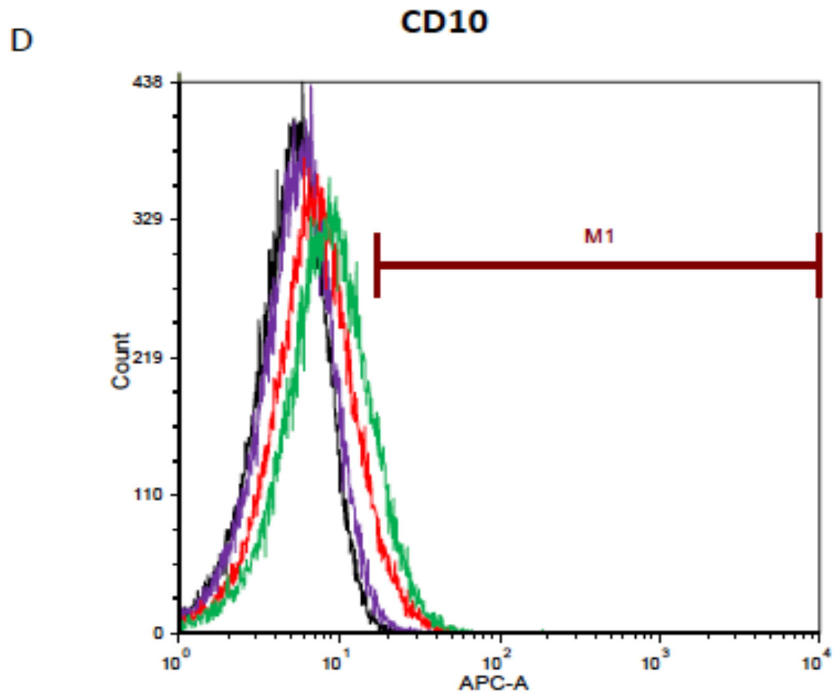

	Filename	Low bound	High bound	# of Events	% of gated	Geometric Mean
(black line)	GUMC-29, Isotype control	17.10	10000.00	184	0.35	21.54
(purple line)	GUMC-30, Isotype control	17.10	10000.00	445	0.88	21.17
(red line)	GUMC-29, CD10	17.10	10000.00	3514	5.86	22.72
(green line)	GUMC-30, CD10	17.10	10000.00	5470	11.57	22.88

Supplementary Figure 1: (*Continued*) Expression of cell surface markers measured by Flow Cytometry. D. CD10. (*Continued*)

E

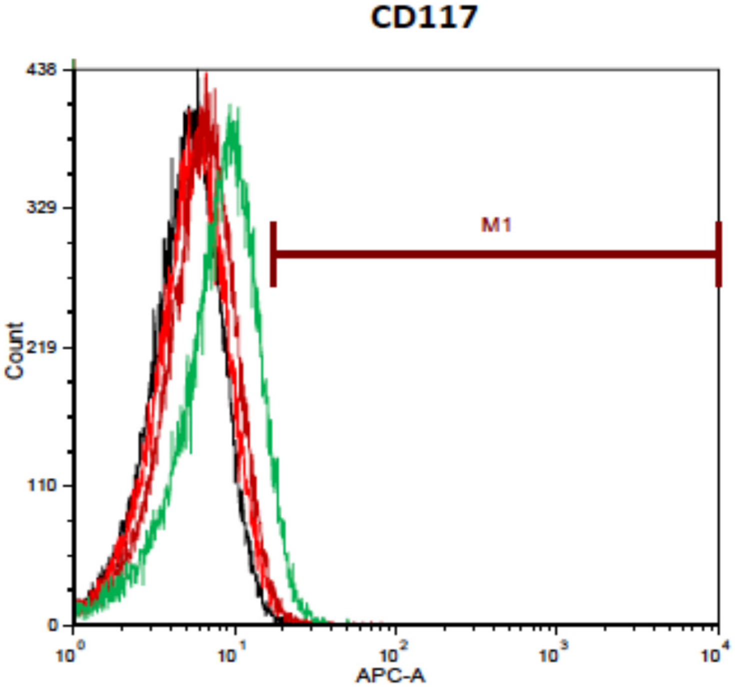

Filename	Low bound	High bound	# of Events	% of gated	Geometric Mean
GUMC-29, Isotype control	17.10	10000.00	184	0.35	21.54
GUMC-30, Isotype control	17.10	10000.00	445	0.88	21.17
GUMC-29, CD117	17.10	10000.00	600	1.09	23.04
GUMC-30, CD117	17.10	10000.00	2544	5.81	24.88

Supplementary Figure 1: (*Continued*) Expression of cell surface markers measured by Flow Cytometry. **E.** CD117. (*Continued*)

F

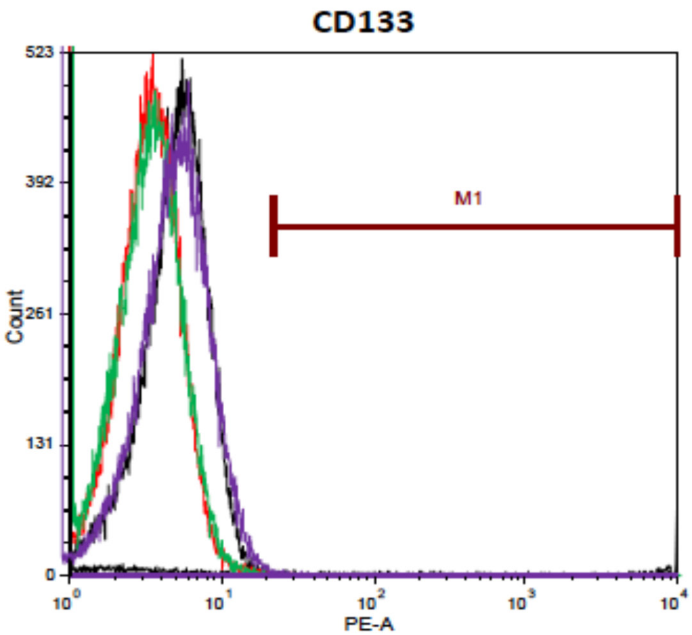

	Filename	Low bound	High bound	# of Events	% of gated	Geometric Mean
(black)	GUMC-29, Isotype control	21.90	10000.00	1037	1.71	812..16
(purple)	GUMC-30, Isotype control	21.90	10000.00	210	0.40	506.96
(red)	GUMC-29, CD133	21.90	10000.00	0.21	0.21	71.98
(green)	GUMC-30, CD133	21.90	10000.00	0.08	0.08	64.08

Supplementary Figure 1: (*Continued*) Expression of cell surface markers measured by Flow Cytometry. F. CD133.

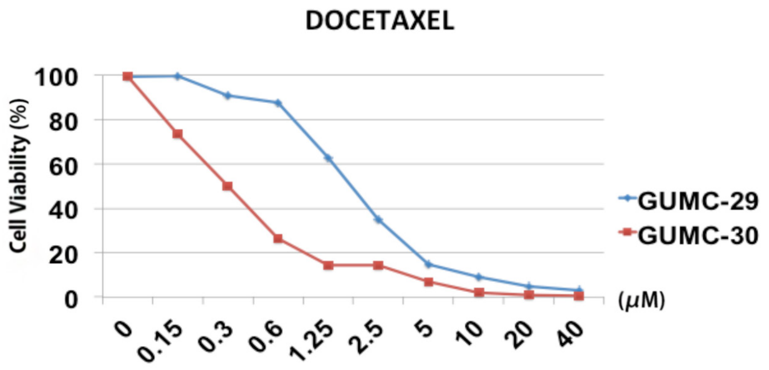

Supplementary Figure 2: Response of CR cells to Docetaxel. CR Cells were plated in triplicate with conditional medium with or without Y-27632 (10 µM) in 96-well microtiter plates (BD Falcon) at 10^3 cells per well. Twenty-four hours later, the cells will be treated with vehicle (dimethylsulfoxide) and docetaxel dissolved in dimethylsulfoxide at various concentrations for 48 hours. Cell viability was measured with the use of the CellTiter-Glo Luminescent Cell Viability Assay (Promega).

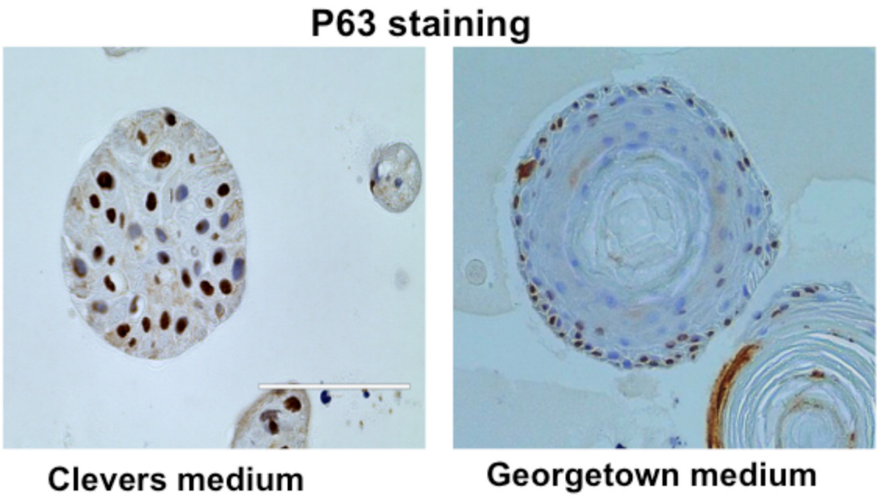

Supplementary Figure 3: Expression of p63 in CR cells when grown in organoid cultures. Organoid cultures were set up according to the protocols described in Materials and Methods. Organoids were fixed overnight with 4% paraformaldehyde at room temperature and the following day with 70% ethanol. Fixed organoids were embedded in paraffin for sectioning at 5 µm before probed with anti-p63 (Santa Cruz, sc25268) for immunohistochemistry. Images of growing organoids were acquired using EVOS FL Cell Imaging System (Invitrogen) for microscopic imaging.

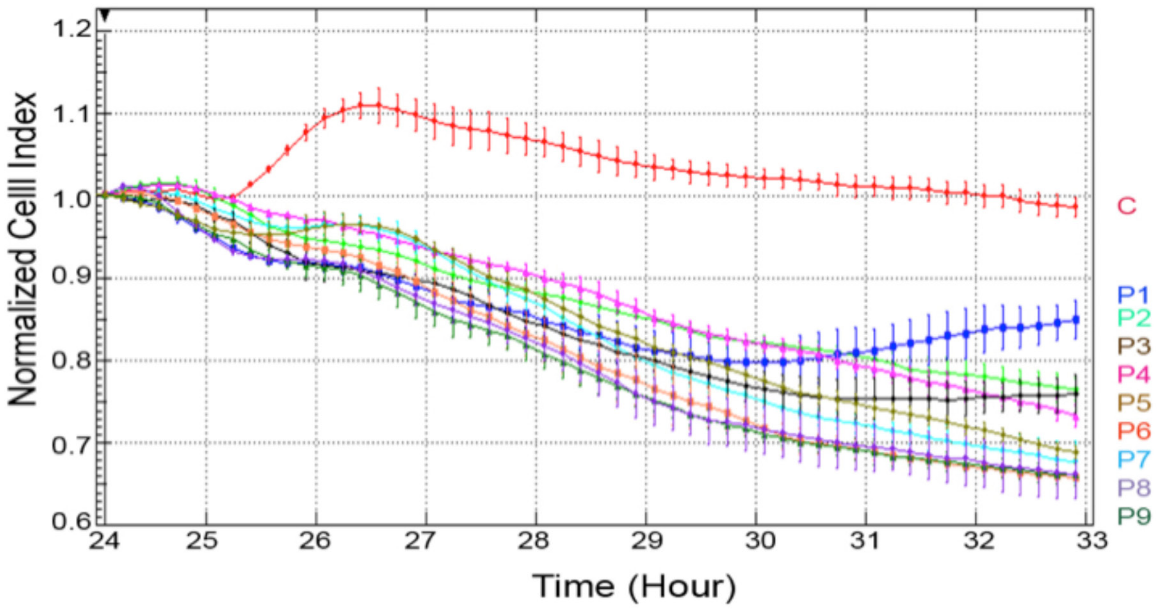

Supplementary Figure 4: Invasion ability of single cell clones from prostate cancer tissue. Single suspension from prostate cancer specimen was serially diluted to 1 cell/200 μ l, then plated to 10 96-well plates with 100 μ l in each well. single cell clones were grown from the prostate cancer culture. The invasive properties of nine individual clones were tested using an xCELLigence RT-CA DP instrument. Briefly, 5 × 10^4 HUVEC cells were seeded on E- plates 16 and allowed to proliferate for 21-24 hours and form an intact monolayer. 1 × 10^4 of tumor CR cells were then added per well and the experiment was run for 2-3 days. The tumor cells showed varying ability to perturb the endothelial monolayer, suggesting that these clones had different ability to invade surrounding tissues.

Supplementary Data Set 1: Gene expression profiling using Affymetrix GeneChip microarrays identified 87 differentially regulated genes in GUMC-30 compared to GUMC-29. See separate Supplementary Data Set 1.

See Supplementary File 1

Supplementary Data Set 2: A total of 815 variants from exome sequencing is only available in the sample GUMC-30. Consensus genotype calls were generated using the UnifiedGenotyper tool from GATK(v3.5) and annotated using the Annovar package. Different basic types of annotations were separated into multiple tables. Impacts are the immediate effects of a variant on gene, transcript, and protein sequences, such as amino acid change, frameshift, or promoter region, etc. (Figure 6A) based on GRCh37 (See separeta Supplementary Data set 2).

See Supplementary File 2

Supplementary Data Set 3: A list of 133 genes common to the two lists was compiled (Figure 5) among which 11 genes belong to the KEGG cancer pathway gene set. See separate Supplementary Data Set 3.

See Supplementary File 3
